# Impact of hemodynamic instability during cytoreductive surgery on survival in high-grade serous ovarian carcinoma

**DOI:** 10.1186/s12885-022-10060-1

**Published:** 2022-09-09

**Authors:** Se Ik Kim, Hyung-Chul Lee, Hyun-Kyu Yoon, Hee Seung Kim, Hyun Hoon Chung, Jae-Weon Kim, Noh Hyun Park, Yong-Sang Song, Maria Lee

**Affiliations:** 1 Department of Obstetrics and Gynecology, Seoul National University College of Medicine, 101 Daehak-Ro, Jongno-Gu, Seoul, 03080 Republic of Korea; 2 Department of Anesthesiology and Pain Medicine, Seoul National University College of Medicine, Seoul, 03080 Republic of Korea; 3 Department of Obstetrics and Gynecology, Seoul National University Hospital, Seoul, 03080 Republic of Korea

**Keywords:** Genital neoplasms, female, Ovarian cancer, High-grade serous carcinoma, Surgery, Hypotension, Blood pressure, Prognosis, Survival outcome

## Abstract

**Background:**

To evaluate the impact of intraoperative hypotension and hemodynamic instability on survival outcomes in patients with high-grade serous ovarian carcinoma (HGSOC).

**Methods:**

We retrospectively identified patients with HGSOC, who underwent primary or interval debulking surgery between August 2013 and December 2019. We collected anesthesia-related variables, including the arterial blood pressure measurements (at 1-min intervals) during the surgery of patients. The cumulative duration of mean arterial blood pressure (MAP) readings under 65 mmHg and two performance measurements (median performance error [MDPE] and wobble) were calculated. We investigated associations between the factors indicating hemodynamic instability and prognosis.

**Results:**

In total, 338 patients were included. Based on the cumulative duration of MAP under 65 mmHg, we divided patients into two groups: ≥30 min and <30 min. The progression-free survival (PFS) was worse in the ≥30 min group (*n* = 107) than the <30 min group (*n* = 231) (median, 18.2 vs. 23.7 months; *P* = 0.014). In multivariate analysis adjusting for confounders, a duration of ≥30 min of MAP under 65 mmHg was identified as an independent poor prognostic factor for PFS (adjusted HR, 1.376; 95% CI, 1.035–1.830; *P* = 0.028). Shorter PFS was observed in the group with a MDPE <−4.0% (adjusted HR, 1.351; 95% CI, 1.024–1.783; *P* = 0.033) and a wobble ≥7.5% (adjusted HR, 1.445; 95% CI, 1.100–1.899; *P* = 0.008). However, no differences were observed in overall survival.

**Conclusion:**

This study suggests that the three intraoperative variables for hemodynamic instability, cumulative duration of MAP <65 mmHg, MDPE, and wobble, might be novel prognostic biomarkers for disease recurrence in patients with HGSOC.

**Supplementary Information:**

The online version contains supplementary material available at 10.1186/s12885-022-10060-1.

## Background

Ovarian cancer, the most lethal gynecologic malignancy, is a global burden with an estimated 313,959 new cases and 207,252 cancer deaths, annually [1]. Ovarian cancer tends to be diagnosed at an advanced stage and has a poor prognosis despite treatment. In the United States, the 5-year survival rate of ovarian cancer was only 49.7% [2]. Among the various types, high-grade serous ovarian carcinoma (HGSOC) is the most common and shows aggressive features [3]. For those with newly diagnosed HGSOC, cytoreductive surgery (CRS) and adjuvant taxane- and platinum-based combination chemotherapy are the established standards of treatment [4].

Optimal cytoreduction is one of the most significant prognostic factors for survival in patients with HGSOC [5]. To achieve no gross residual disease or complete cytoreduction, gynecologic oncologists perform CRS with maximal effort [6]. Such aggressive surgery involves long operative times and excessive bleeding, especially in those with a widespread, high tumor burden. Therefore, intraoperative hemodynamic instability is frequently observed in patients with HGSOC.

Intraoperative hemodynamic instability, defined as an abnormal or unstable blood pressure that causes inadequate blood flow to the organ (i.e., perfusion failure), is known to be associated with increased postoperative complications and mortality in various surgeries [7]. In non-cardiac surgeries, intraoperative hypotension significantly increases the risk of postoperative acute kidney injury [8, 9], myocardial injury [9, 10], and mortality [11]. In addition to monitoring for intraoperative hypotension, performance measures that evaluate deviations in blood pressure, such as median performance error (MDPE) and wobble, can be applied in clinical practice to evaluate hemodynamic instability [12, 13]. MDPE indicates the percentage of the median intraoperative blood pressure that is lower or higher than preoperative ward blood pressure. Wobble indicates the cumulative deviation of blood pressure from the reference value of the individual’s intraoperative blood pressure. Both MDPE and wobble are relative and individual blood pressure indices that can be measured and monitored during surgery.

To date, very few studies have investigated the relationship between intraoperative hypotension and survival outcomes in a malignant disease [14, 15]. However, the indicators of intraoperative hemostatic instability that have prognostic roles in patients with HGSOC remain unknown. Thus, this study aimed to investigate the impact of hemodynamic instability during CRS on survival outcomes in patients with HGSOC.

## Methods

### Study population

From the Ovarian Cancer Cohort Database, we searched patients who met the following inclusion criteria: (1) patients older than 18 years of age; (2) diagnosed with International Federation of Gynecology and Obstetrics (FIGO) stage IC-IV HGSOC and primarily treated at our institutional hospital; (3) those who underwent CRS, either primary debulking surgery (PDS) or interval debulking surgery after neoadjuvant chemotherapy (NAC), aimed at removing all macroscopic tumors between August 2013 and December 2019; and (4) those whose electronic anesthetic records including blood pressure data (at 1-min intervals) during surgery were available. However, patients with the following conditions were excluded: (1) patients with any malignancy other than HGSOC; (2) those who were enrolled in first-line clinical trials incorporating therapeutic agents, such as immune checkpoint inhibitors and poly (ADP-ribose) polymerase inhibitors, because they may influence patients’ recurrence rate; and (3) those with insufficient clinicopathologic data or who were lost to follow-up during primary treatment (Supplementary Fig. S1).

### Data collection

From the electronic medical records of patients, we collected the clinicopathologic data including age at diagnosis, body mass index (BMI), comorbidities, American Society of Anesthesiologists (ASA) score, FIGO stage, initial serum CA-125 levels, administration of NAC, and maximal size of residual tumor after CRS. Surgical complexity scores were calculated based on the number and relative difficulty of the procedures performed, according to Aletti et al. [16]. We also collected anesthesia-related data, such as operative times and estimated blood loss (EBL).

After surgery, all patients received taxane- and platinum-based chemotherapy as part of the primary treatment. During surveillance, computed tomography scans were routinely performed once every 3 to 4 months for the first 2 years, once every 6 months for the next 2 years, and annually thereafter, or earlier when examination findings or symptoms were suspicious for recurrence. In terms of survival outcomes, we defined progression-free survival (PFS) as the time interval between the date of treatment initiation and the date of disease progression or recurrence confirmed by the Response Evaluation Criteria in Solid Tumours version 1.1 [17] or the Gynecologic Cancer InterGroup CA-125 criteria [18]. Overall survival (OS) was defined as the time interval between the date of diagnosis and the date of cancer-related death or last visit.

### Hemodynamic instability variables

We defined preoperative systolic blood pressure (SBP_ward_) as the median systolic blood pressure measured in the general ward the day before surgery. Intraoperative systolic blood pressure (SBP_k_) was defined as the invasive arterial systolic blood pressure measurements documented during surgery, obtained from the electronic anesthetic record (collected at 1-min intervals and averaged at 5-min intervals). We defined intraoperative hypotension as mean arterial pressure (MAP) below the absolute threshold of 65 mmHg. The cumulative duration of the MAP less than 65 mmHg was calculated in each patient. The MDPE, representing the median relative blood pressure difference between the intraoperative and preoperative values, and the wobble, representing hemodynamic instability during surgery, were also calculated using the following formulae, where k represented the index for intraoperative blood pressure measurement [13].

$$MDPE\left(\%\right)= median\ \left(\frac{SB{P}_k- SB{P}_{ward}}{SB{P}_{ward}}\right)\times 100$$

$$Wobble\left(\%\right)= median\ \left(\left| MDPE-\frac{SB{P}_k- SB{P}_{ward}}{SB{P}_{ward}}\times 100\right|\right)$$

$$k=1,2,3,\dots, \mathrm{N}\ \left(\mathrm{N},\mathrm{number}\ \mathrm{of}\ \mathrm{intraoperative}\ \mathrm{blood}\ \mathrm{pressure}\ \mathrm{measurement}\right)$$

**Fig. 1:**
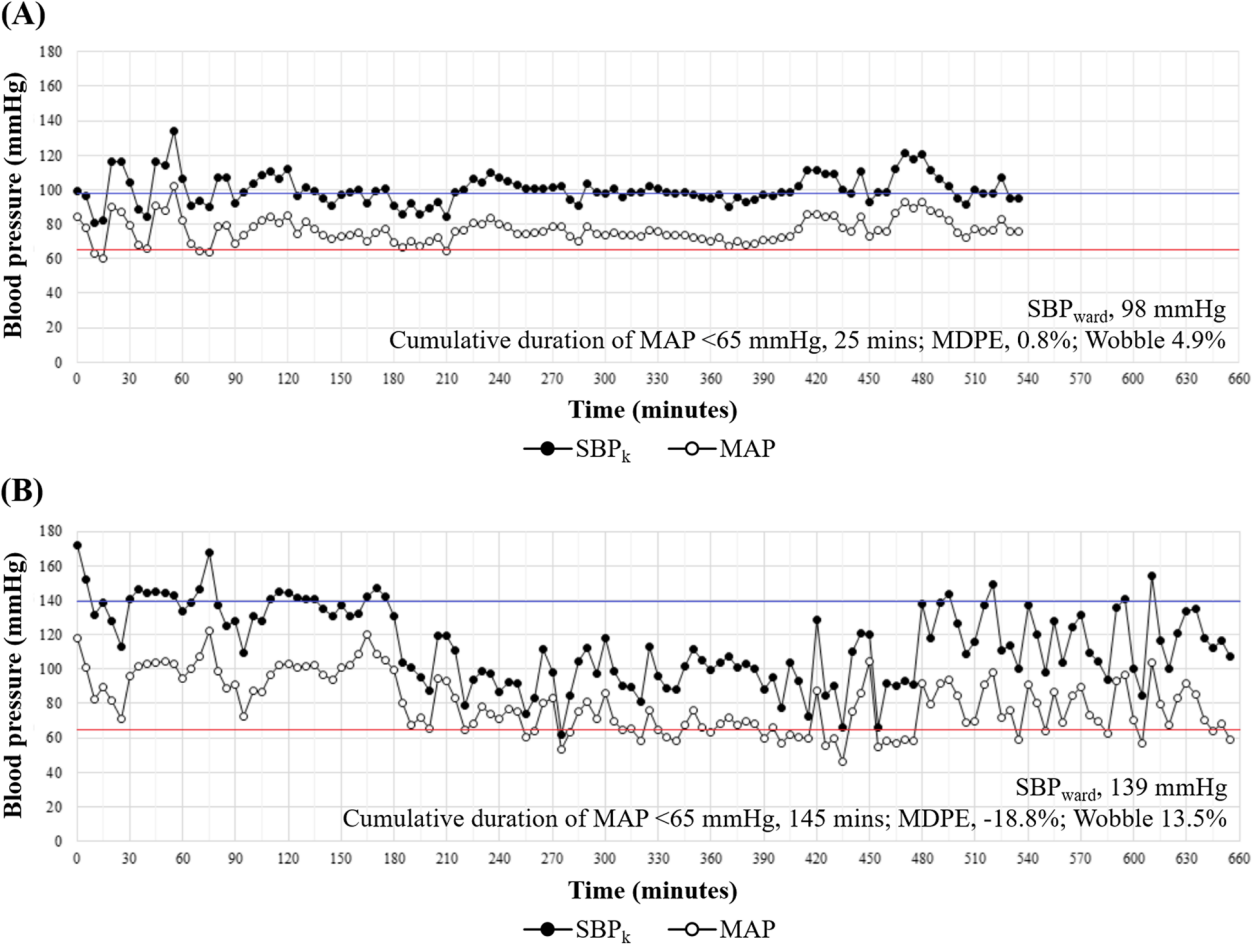
Representative images of intraoperative blood pressure monitoring and the values for the three variables indicating hemodynamic instability. The red line indicates the 65 mmHg mark while the blue line indicates the median systolic blood pressure measured in the general ward the day before surgery (SBP_ward_). Black dots indicate intraoperative systolic blood pressure (SBP_k_), obtained from the electronic anesthetic record (collected at 1-min intervals and averaged at 5-min intervals). White dots indicate intraoperative mean arterial pressure (MAP). **A** A 62-year-old woman with FIGO stage IIIC HGSOC. After an operative time of 8.1 h for primary debulking surgery (anesthesia time: 9.2 h), complete cytoreduction was achieved. **B** A 71-year-old woman with FIGO stage IVB HGSOC. After an operative time of 9.9 h for primary debulking surgery (anesthesia time: 11.1 h), complete cytoreduction was achieved

Representative images for intraoperative blood pressure monitoring and the calculated three hemodynamic instability variables are depicted in Fig. 1.

### Statistical analysis

Patients were divided into two groups by the cumulative duration of MAP under 65 mmHg, MDPE, and wobble, using the mean values or values close to the mean for clinical utilization. Then, patients’ clinicopathologic characteristics and survival outcomes were compared between the two groups. We used the Student’s *t* or Mann–Whitney *U* test for continuous variables, and Pearson’s chi-squared or Fisher’s exact test for categorical variables. For survival analyses, we used the Kaplan–Meier method with log-rank test and the Cox proportional hazards regression models to calculate the adjusted hazard ratios (HRs) and 95% confidence intervals (CIs). These statistical analyses were performed using SPSS software (version 25.0; IBM Corp., Armonk, NY, USA). For correlation analyses, we conducted Pearson’s correlation coefficient test and calculated correlation values, using GraphPad Prism 5 software (GraphPad Inc., La Jolla, CA, USA). A *P*-value < 0.05 was considered statistically significant.

## Results

### Study population

In total, 338 patients were included in this analysis. Table 1 depicts the clinicopathologic characteristics of patients. Among the study population, 85.8% had stage III-IV disease. Two-thirds of patients (66.6%) underwent PDS, while the other one-third (33.4%) underwent NAC followed by interval debulking surgery. The median surgical complexity score was 6 [interquartile range (IQR), 4–9] and complete cytoreduction was achieved in 74.9% of the patients.

**Table 1:** Clinicopathologic characteristics of all patients

Characteristics	All (*n* = 338, %)
***At the time of diagnosis***	
FIGO stage	
IC	19 (5.6)
II	29 (8.6)
III	169 (50.0)
IV	121 (35.8)
Initial serum CA-125 ^a^, IU/ml	
Median (IQR)	878.0 (289.0–2433.0)
Primary treatment strategy	
Primary debulking surgery	225 (66.6)
Neoadjuvant chemotherapy	113 (33.4)
***At the time of surgery***	
Age, years	
Mean ± SD	57.8 ± 11.2
BMI, kg/m^2^	
Median (IQR)	23.2 (20.9–25.4)
Underweight (<18.5)	24 (7.1)
Normal (18.5–22.9)	134 (39.6)
Overweight (23.0–24.9)	81 (24.0)
Obesity (≥25.0)	99 (29.3)
Comorbidities	
Hypertension	58 (17.2)
Diabetes	19 (5.6)
Liver disease	7 (2.1)
Heart disease	9 (2.7)
Renal disease	2 (0.6)
Vascular disease	2 (0.6)
Neurologic disease	5 (1.5)
Asthma	2 (0.6)
ASA classification	
1	65 (19.2)
2	220 (65.1)
3	52 (15.4)
4	1 (0.3)
Surgical complexity score	
Median (IQR)	6 (4–9)
Low (≤3)	33 (9.8)
Intermediate (4–7)	175 (51.8)
High (≥8)	130 (38.5)
Residual tumor after PDS/IDS	
Complete cytoreduction (R0)	253 (74.9)
< 1 cm	48 (14.2)
1–2 cm	22 (6.5)
≥ 2 cm	15 (4.4)

*Abbreviations*: *ASA* American Society of Anesthesiologists, *BMI* Body mass index, *CA-125* Cancer antigen 125, *FIGO* International Federation of Gynecology and Obstetrics, *IDS* Interval debulking surgery, *IQR* Interquartile range, *SD* Standard deviation

Missing data: ^a^ 3

The perioperative characteristics of patients are presented in Table 2. The median anesthesia and operative durations were 5.9 h (IQR, 4.5–7.7) and 4.8 h (IQR, 3.3–6.5), respectively. In regard to the variables related to hemodynamic instability, the mean values of the cumulative duration of MAP <65 mmHg, MDPE, and wobble were 27.0 min, −4.4, and 7.6%, respectively. Thus, 30.0 min, −4.0, and 7.5% were set as cut-off values based on which the study population was divided into groups.

**Table 2:** Perioperative characteristics of all patients

Variables	All (*n* = 338)	
	Mean ± SD	Median (IQR)
Preoperative Hb, g/dl	11.9 ± 1.4	11.9 (10.8–12.8)
Anesthesia time, h	6.2 ± 2.4	5.9 (4.5–7.7)
Operative time, h	5.1 ± 2.4	4.8 (3.3–6.5)
Urine output ^a^, ml	600.4 ± 560.0	450.0 (250.0–750.0)
Estimated blood loss ^b^, ml	1432.0 ± 1690.5	900.0 (500.0–1825.0)
RBC transfusion, pack	2.5 ± 3.5	1 (0–4)
Fluid infusion ^c^, ml	4466.9 ± 2934.2	3800.0 (2337.5–5700.0)
Colloid	426.5 ± 500.8	275.0 (0–712.5)
Crystalloid ^c^	4024.7 ± 2669.6	3400.0 (2100.0–5100.0)
MAP under 65 mmHg, min	27.0 ± 37.6	15.0 (5.0–35.0)
MDPE (%)	−4.4 ± 11.4	−5.1 (−12.0–3.1)
Wobble (%)	7.6 ± 2.3	7.3 (6.0–9.1)

*Abbreviations*: *Hb* Hemoglobin, *SD* Standard deviation, *RBC* Red blood cell, *IQR* Interquartile range, *MAP* Mean arterial blood pressure, *MDPE* Median performance error

Missing data: ^a^ 23; ^b^ 21; ^c^ 12

**Fig. 2:**
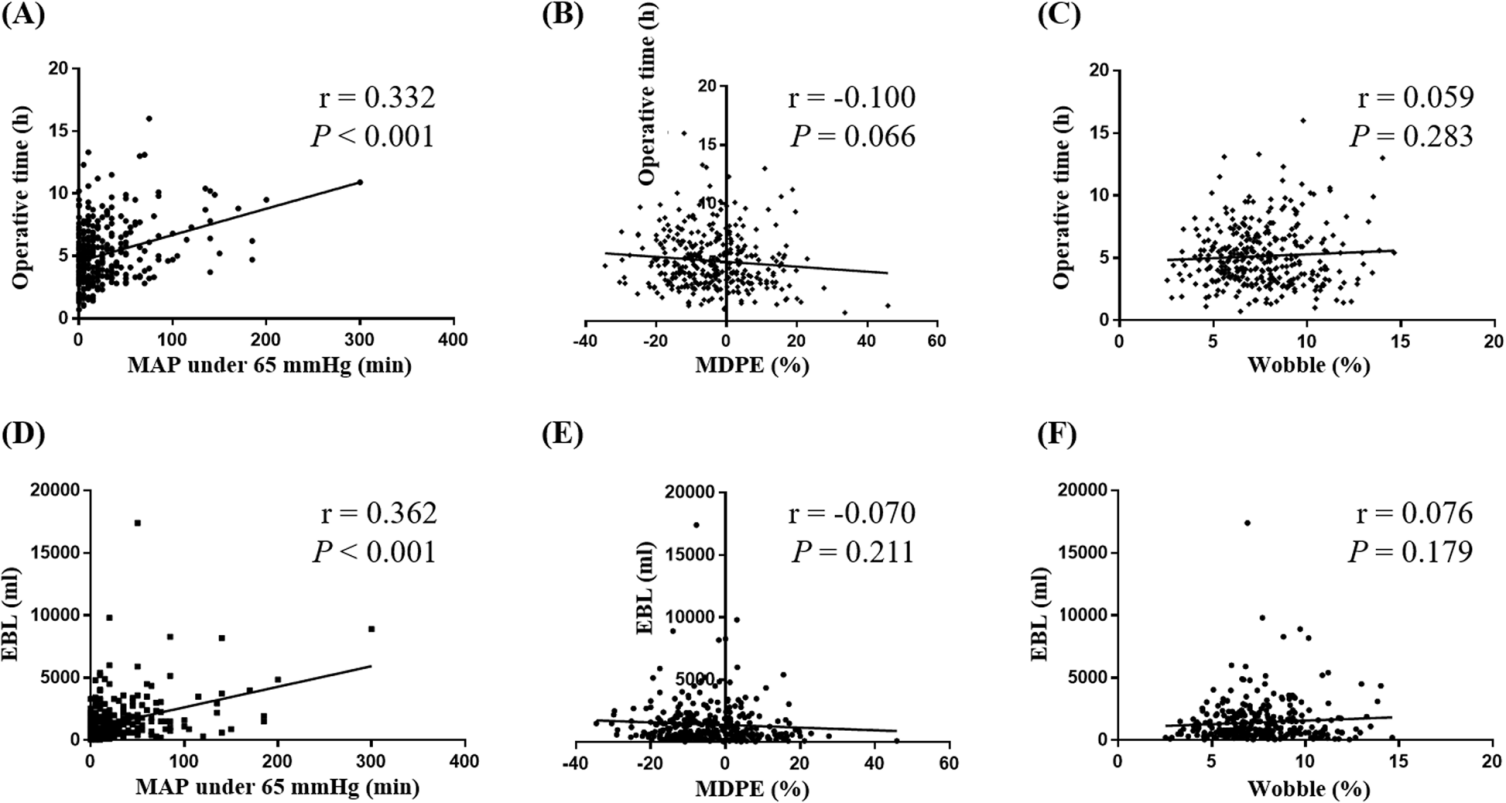
Correlations between the three variables indicating hemodynamic instability and operative time (Upper) and estimated blood loss (Lower). **A**, **D** Cumulative duration of MAP <65 mmHg; (**B**, **E**) Median performance error; (**C**, **F**) Wobble

We investigated the correlations between operative time and the three variables indicating hemodynamic instability (Fig. 2A-C). Although the cumulative duration of MAP under 65 mmHg was significantly correlated with the operative time, but the relationship was weak (Pearson’s correlation coefficient r = 0.332; *P* < 0.001). There were no correlations between operative time and either MDPE or wobble. We also investigated the correlations between EBL and the three variables indicating hemodynamic instability variables (Fig. 2D-F). Among the three variables, only cumulative duration of MAP under 65 mmHg was significantly correlated with EBL; a weak, positive correlation was observed between the two (r = 0.362; *P* < 0.001).

### Analysis based on the cumulative duration of MAP <65 mmHg

Based on the cumulative duration of MAP <65 mmHg, 107 (31.7%) and 231 (68.3%) were assigned to ≥30 min and <30 min groups, respectively. Patients in the ≥30 min group were significantly older (*P* = 0.001) and had a higher prevalence of diabetes (*P* = 0.011) and greater serum CA-125 levels (median, 1438.0 vs. 794.5 IU/ml; *P* = 0.012), compared to those in the <30 min group (Supplementary Table S1). No differences were observed in FIGO stage and proportion of NAC between the groups. However, the ≥30 min group had significantly higher surgical complexity scores (median, 8 vs. 5; *P* < 0.001). Nevertheless, the two groups showed similar residual tumor sizes after CRS (*P* = 0.771).

In terms of perioperative characteristics, patients in the ≥30 min group had significantly longer operative times (median, 5.5 vs. 4.3 h; *P* < 0.001) and higher EBL (median, 1500.0 vs. 675.0 ml; *P* < 0.001), compared to those in the <30 min group (Supplementary Table S2). Amount of packed red blood cell transfusions and fluid infusions was also higher in the ≥30 min group.

**Fig. 3:**
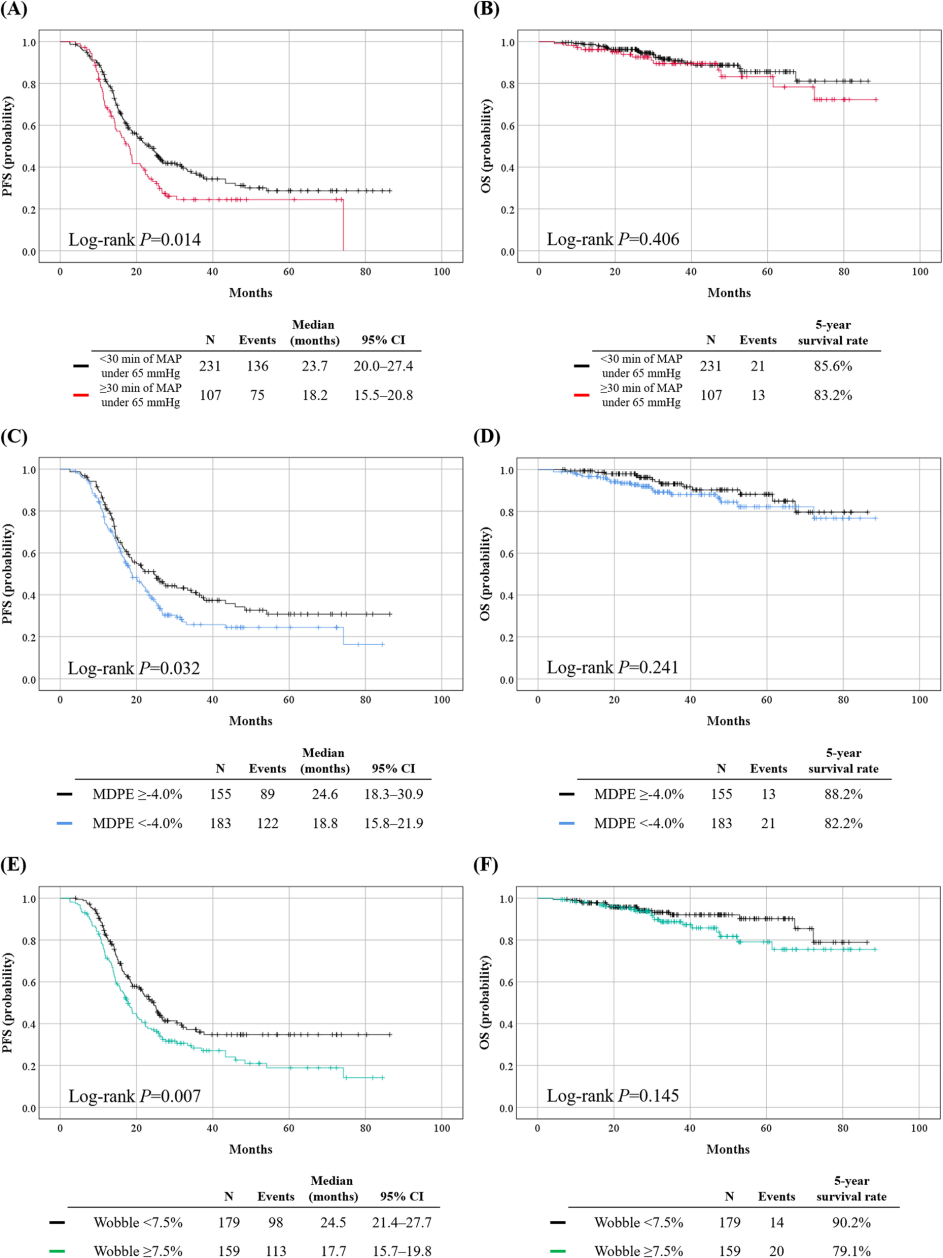
Survival outcomes in patients classified according to the cumulative duration of MAP <65 mmHg (Upper); Median performance error (Middle); and wobble (Lower). **A**, **C**, **E** Progression-free survival; (**B**, **D**, **F**) Overall survival

During the median observation period of 32.8 months, 211 (62.4%) patients experienced disease recurrence and 34 (10.1%) patients died of the disease. In survival analyses, patients in the ≥30 min group showed significantly shorter PFS than the <30 min group (median, 18.2 vs. 23.7 months; *P* = 0.014), but similar OS (5-year survival rate, 83.2% vs. 85.6%; *P* = 0.406) (Fig. 3A, B). In multivariate analyses adjusting for FIGO stage, NAC, operative time, and residual tumor status after surgery, ≥30 min of MAP under 65 mmHg was identified as an independent poor prognostic factor for PFS (adjusted HR, 1.376; 95% CI, 1.035–1.830; *P* = 0.028) (Table 3), while it did not affect OS (Supplementary Table S3).

**Table 3:** Factors associated with patients’ progression-free survival

Variables	*Univariate analysis*		*Multivariate analysis*							
	HR (95% CI)	*P*	aHR (95% CI)	*P*	aHR (95% CI)	*P*	aHR (95% CI)	*P*	aHR (95% CI)	*P*
FIGO stage										
III-IV (*n* = 290) vs. I-II (*n* = 48)	4.973 (2.631–9.399)	< 0.001	2.729 (1.397–5.331)	0.003	2.915 (1.494–5.687)	0.002	2.933 (1.504–5.720)	0.002	2.913 (1.494–5.677)	0.002
Neoadjuvant chemotherapy										
Yes (*n* = 113) vs. No (*n* = 225)	2.160 (1.639–2.845)	< 0.001	2.107 (1.572–2.823)	< 0.001	2.003 (1.497–2.680)	< 0.001	1.973 (1.471–2.646)	< 0.001	1.994 (1.489–2.671)	< 0.001
Operative time, h										
≥5.0 (*n* = 161) vs. <5.0 (*n* = 177)	1.477 (1.125–1.938)	0.005	1.331 (1.003–1.767)	0.048	1.325 (0.998–1.759)	0.052	1.358 (1.023–1.802)	0.034	1.392 (1.048–1.849)	0.022
Residual tumor after surgery										
Residual (*n* = 85) vs. R0 (*n* = 253)	2.555 (1.897–3.442)	< 0.001	2.333 (1.719–3.168)	< 0.001	2.323 (1.713–3.150)	< 0.001	2.267 (1.671–3.076)	< 0.001	2.279 (1.681–3.091)	< 0.001
MAP under 65 mmHg, min										
Continuous (*n* = 338)	1.002 (0.999–1.005)	0.289								
≥30.0 (*n* = 107) vs. <30.0 (*n* = 231)	1.423 (1.073–1.887)	0.014	1.376 (1.035–1.830)	0.028						
MDPE (%)										
Continuous (*n* = 338)	0.993 (0.981–1.005)	0.244								
<−4.0 (*n* = 183) vs. ≥−4.0 (*n* = 155)	1.349 (1.025–1.775)	0.032			1.351 (1.024–1.783)	0.033				
Wobble (%)										
Continuous (*n* = 338)	1.096 (1.032–1.165)	0.003					1.082 (1.018–1.150)	0.012		
≥7.5 (*n* = 159) vs. <7.5 (*n* = 179)	1.449 (1.106–1.900)	0.007							1.445 (1.100–1.899)	0.008

*Abbreviations*: *aHR* Adjusted hazard ratio, *BMI* Body mass index, *CI* Confidence interval, *FIGO* International Federation of Gynecology and Obstetrics, *HR* Hazard ratio, *MAP* Mean arterial blood pressure, *MDPE* Median performance error, *R0* Complete cytoreduction

### Analysis based on the median performance error

Among the study population, 183 (54.1%) and 155 (45.9%) were assigned to MDPE <−4.0% and ≥−4.0% groups, respectively. Patients in the MDPE <−4.0% group were significantly older (*P* = 0.002) and had higher BMI (*P* = 0.001), compared to those in the MDPE ≥−4.0% group (Supplementary Table S4). Other characteristics, such as FIGO stage, proportion of NAC, surgical complexity score, and residual tumor size after surgery, were similar between the groups. In terms of perioperative characteristics, operative duration, EBL, and amount of packed red blood cell transfusion was similar. However, amount of infused crystalloid fluid was significantly higher in the MDPE <−4.0% group (*P* = 0.038) (Supplementary Table S2).

In survival analyses, the MDPE <−4.0% group showed significantly shorter PFS than the ≥−4.0% group (median, 18.8 vs. 24.6 months; *P* = 0.032), but similar OS (5-year survival rate, 82.2% vs. 88.2%; *P* = 0.241) (Fig. 3C, D). Multivariate analyses adjusting for clinicopathologic factors revealed that the PFS significantly deteriorated in patients from the MDPE <−4.0% (adjusted HR, 1.351; 95% CI, 1.024–1.783; *P* = 0.033) (Table 3), but was not associated with patients’ OS (Supplementary Table S3).

### Analysis based on wobble

Among the study population, 159 (47.0%) and 179 (53.0%) were assigned to wobble ≥7.5 and <7.5% groups, respectively. Patients in the wobble ≥7.5% group were significantly older (*P* < 0.001) and had higher prevalence of diabetes (*P* = 0.004), compared to the wobble <7.5% group (Supplementary Table S5). Although the two groups had similar serum CA-125 levels and FIGO stage, proportion of NAC was significantly higher in the wobble ≥7.5% group (40.3% vs. 27.4%; *P* = 0.012). Nevertheless, there were no differences in surgical complexity scores and residual tumor sizes after surgery between the groups. The two groups had similar perioperative characteristics, except preoperative hemoglobin, which was significantly lower in the wobble ≥7.5% group (*P* = 0.009) (Supplementary Table S2).

In survival analyses, significantly shorter PFS was observed in the wobble ≥7.5% group compared to the wobble <7.5% group (median, 17.7 vs. 24.5 months; *P* = 0.007). However, no differences were observed in OS (5-year survival rate, 79.1% vs. 90.2%; *P* = 0.145) (Fig. 3E, F). Multivariate analyses revealed that a wobble ≥7.5% affected the PFS of patients adversely (adjusted HR, 1.445; 95% CI, 1.100–1.899; *P* = 0.008) (Table 3), but it was not associated with patients’ OS (Supplementary Table S3). Regarded as a continuous variable, an increase in wobble was associated with a shortening in the PFS (adjusted HR, 1.082; 95% CI, 1.018–1.150; *P* = 0.012) (Table 3), but not OS (Supplementary Table S3).

## Discussion

In this study, we investigated the prognostic impact of three intraoperative variables indicating hemodynamic instability: cumulative duration of MAP <65 mmHg, MDPE, and wobble, on survival outcomes in patients with HGSOC who underwent CRS. The results identified a MAP under 65 mmHg for ≥30 min, MDPE <−4.0%, and wobble ≥7.5% as poor prognostic factors for PFS. In contrast, these factors did not affect patients’ OS.

HGSOC is one of the few epithelial cancers, in which the removal of metastatic tumors has been found to improve survival outcomes [5, 6]. In reality, despite the best efforts by gynecologic oncologists to achieve complete cytoreduction, patients with HGSOC experience recurrence several times, develop chemoresistance, and succumb to the disease. Therefore, it is very important to discover novel prognostic factors in these patients. Based on our study results, if patients have even one of the three hemodynamic instability factors, they are identified at high risk of disease recurrence, and physicians may recommend a more aggressive treatment and intensive surveillance with frequent measurements of serum CA-125 levels and imaging studies.

In the current study, we chose the three variables indicating hemodynamic instability for the following reasons: the cumulative duration of MAP <65 mmHg is an absolute, standardized blood pressure indicator. In addition, we considered the MDPE as a comparative, individualized blood pressure indicator. Lastly, wobble was selected as it represented blood pressure instability during surgery. Since these variables have not yet been evaluated in various cancer surgeries in earnest, we have referred to the previous studies conducted in patients with benign diseases [12, 13].

Intraoperative hypotension is common during non-cardiac surgery. According to a sub-study of the POISE-2, a 10,010-patient factorial-randomized trial of aspirin and clonidine for prevention of myocardial infarction, 34.9% of the patients experienced intraoperative hypotension [19]. As intraoperative hypotension is associated with an increase in postoperative mortality and morbidity [8–11, 20], an expert consensus recommends that intraoperative blood pressure be maintained above 100 mmHg for systolic blood pressure and above 60–70 mmHg for MAP during elective, non-cardiac surgery [7].

Similar to our study, van Waes et al. calculated the cumulative duration of MAP under 60 mmHg during vascular surgery in older patients, and reported that >30 min of MAP under 60 mmHg was significantly associated with myocardial injury (relative risk, 1.7; 98.8% CI, 1.1–2.6; *P* = 0.004) [10]. Consistent results were also observed when different definitions of hypotension were used (i.e., more than 30 min of ≥30% or ≥40% decrease from baseline MAP) [10]. Meanwhile, a retrospective cohort study by Salmasi et al. also reported that prolonged exposure of MAP under 65 mmHg during non-cardiac surgery increased the odds of both myocardial and kidney injury [9]. These studies highlight the degree and duration of intraoperative hypotension, both of which are important in the development of postoperative complications.

Regarding primary cancer surgery, Huang et al. investigated the impact of intraoperative hypotension on survival outcomes in 676 patients who underwent lung cancer surgery [14]. The authors reported that intraoperative hypotension, defined as a systolic blood pressure <100 mmHg for at least 5 min, was significantly associated with poorer OS (adjusted HR, 1.382; 95% CI, 1.047–1.825; *P* = 0.023). This study suggested that survival outcomes might be worsened even with a short duration of intraoperative hypotension. In an earlier retrospective study by Younes et al., comprising 116 patients who underwent complete hepatic resection for colorectal metastases, the number of intraoperative hypotension episodes, defined as a ≥ 20% decrease from baseline MAP, was significantly associated with shorter PFS [15]. Despite differences in the study populations and definitions of hypotension in the studies, our study results are consistent with those in previous studies.

Intraoperative hypotension can also be measured by performance measurements, MDPE and wobble, both of which are relatively new indicators for hemodynamic instability. In the previous studies conducted by our research team, MDPE was found to be associated with a 30-day and overall mortality after cardiac surgery performed using cardiopulmonary bypass [12], and wobble was observed to be associated with mortality after liver transplantation [13]. In the current study, we identified additional roles for the performance measurements: MDPE <−4.0% and wobble ≥7.5% were independent poor prognostic factors for PFS in patients with HGSOC, who received CRS. Herein, we propose that both MDPE and wobble are useful biomarkers that can be employed to quantify the degree of intraoperative hypotension and to predict survival outcomes following CRS. While MDPE measures the degree of hypotension, wobble measures blood pressure variability and detects concealed or treated hypotension. Furthermore, the real-time intraoperative measurement of MDPE and wobble is possible, enabling anesthesiologists to provide adequate, timely hemodynamic management by the use of inotropic agents, vigorous fluid resuscitation, or transfusion.

In our study, MAP under 65 mmHg for ≥30 min, MDPE <−4.0%, and wobble ≥7.5%, were associated with deteriorated PFS. The negative value of MDPE indicates a lower intraoperative SBP than the preoperative SBP_ward_, presumably caused by general anesthesia, blood loss, or hypovolemia. Patients with MDPE <−4.0% were more likely to experience MAP under 65 mmHg for ≥30 min (*P* = 0.001) and to have wobble ≥7.5% (*P* = 0.015). Therefore, intraoperative hypotension seems to play a major role in increased disease recurrence rate. We hypothesize that hypoxia is responsible for the increased recurrence rate in HGSOC patients who experienced intraoperative hemodynamic instability. Hypoxia is known as a key pro-cancerous feature of the tumor microenvironment. As described by Huang et al. [14], intraoperative hypotension could aggravate a hypoxic tumor microenvironment, inducing the development of aggressive phenotypes of tumors by overexpression of hypoxia-inducible factor-1 (HIF-1) leading to cancer progression and dissemination [21, 22]. Recent evidence also suggests that non-coding RNAs, shuttled via exosomes, regulate cancer biology, and reshape the hypoxic tumor microenvironment [23]. Intraoperative hypotension-induced hypoxia could also promote systemic inflammation, which is known to have pro-tumorigenic effects [24, 25]. Considering the fact that some gene expression and activation of signaling pathways occur in a very short time [26], even a transient exposure to hypoxia during surgery may lead to such changes.

Some might argue that such adverse effects from intraoperative hypotension would not exist in patients who achieved complete cytoreduction as they do not have any gross residual tumor. In the study population, 74.9% achieved complete cytoreduction. However, cancer stem cells within the surgically resected tumor bed might have survived and been affected by intraoperative hypotension-induced hypoxia [27]. The remaining cancer stem cells might have evolved toward drug resistance [28, 29]. Despite adjuvant chemotherapy, resistant cancer stem cells might survive and consequently affect the disease progression in patients [30, 31].

Interestingly, none of the three intraoperative variables for hemodynamic instability affected OS in the current study. The possible explanations for their not affecting OS are as follows: first, the observation period might be relatively short to observe death events. Second, approximately 10% of the relapsed were lost to follow-up within 6 months after the confirmation of the first recurrence. Third, although we did not investigate detailed information on treatment methods for recurrent HGSOC, some might be cured or salvaged by second-line treatment. Fourth, physicians at our institutions have actively enrolled those who recurred to phase III randomized controlled trials, such as GOG-213, DEKSTOP-III, and SOLO2, or other clinical trials, which might affect post-progression survival. Lastly, hypoxic tumor microenvironments induced by intraoperative hypotension might be altered or further evolve during the course of treatments and disease recurrences [32].

Based on the results of our study, we emphasize the clinical importance of monitoring intraoperative blood pressure and preventing hypotension and hemodynamic instability during CRS. The cumulative duration of MAP <65 mmHg, MDPE, and wobble, are potentially modifiable factors. To ensure the probability of cure and improved PFS of patients with HGSOC, intraoperative hemodynamic instability should be avoided and corrected promptly by adequate transfusion or intravenous fluid infusion, and use of drugs, such as vasopressors and inotropes.

The current study has some limitations. First, there might be selection bias or other inherent issues owing to the retrospective study design. Second, although 338 patients were included, a larger sample size may be needed for generalization of the results. Third, while calculating the cumulative duration of MAP <65 mmHg, we did not distinguish patients who developed continuous intraoperative hypotension and those who crossed the threshold repeatedly. Fourth, as survival for ovarian cancer is known to be affected by hospital volume and quality of care [33], inconsistent results might be observed in other study populations. Further large, multi-center cohort studies are warranted to determine more precise cut-off values of the three intraoperative variables for hemodynamic instability and to validate our findings. By combining hemodynamic instability variables, clinical factors, and laboratory test results [34], we might be able to develop models predicting prognosis after primary treatment for clinical utility. Despite these limitations, the current study included selected patients using strict inclusion/exclusion criteria, and investigated the prognostic impact of hemodynamic instability in those with HGSOC for the first time.

## Conclusions

In conclusion, our study results suggest that hemodynamic instability during CRS significantly might influence HGSOC patients’ PFS. The three intraoperative variables for hemodynamic instability, cumulative duration of MAP <65 mmHg, MDPE, and wobble, might be novel prognostic biomarkers for disease recurrence as well as surrogates for other poor prognostic factors. This study promotes the importance of thorough blood pressure monitoring and employment of active and immediate countermeasures by anesthesiologists who attend CRS.

## Supplementary Information

**Additional file 1: Supplementary Fig. S1.** Flow diagram depicting the selection of the study population.

**Additional file 2: Supplementary Table S1.** Clinicopathologic characteristics in patients classified according to the cumulative duration of MAP <65 mmHg.

**Additional file 3: Supplementary Table S2.** Perioperative characteristics in patients classified according to the cumulative duration of MAP <65 mmHg, median performance error, and wobble.

**Additional file 4: Supplementary Table S3.** Factors associated with patients’ overall survival.

**Additional file 5: Supplementary Table S4.** Clinicopathologic characteristics in patients classified according to the median performance error.

**Additional file 6: Supplementary Table S5.** Clinicopathologic characteristics in patients classified according to wobble.

## Data Availability

The datasets used and/or analyzed during the current study are available from the corresponding author on reasonable request.

## Abbreviations

ASA: American Society of Anesthesiologists
BMI: Body mass index
CI: Confidence interval
CRS: Cytoreductive surgery
EBL: Estimated blood loss
FIGO: International Federation of Gynecology and Obstetrics
HGSOC: High-grade serous ovarian carcinoma
HR: Hazard ratio
IQR: Interquartile range
MAP: Mean arterial blood pressure
MDPE: Median performance error
NAC: Neoadjuvant chemotherapy
OS: Overall survival
PDS: Primary debulking surgery
PFS: Progression-free survival
SBP: Systolic blood pressure
