# Supplementary figures and images for: Impact of hemodynamic instability during cytoreductive surgery on survival in high-grade serous ovarian carcinoma
Source: BMC Cancer. 2022 Sep 9;22:965. doi: 10.1186/s12885-022-10060-1 (PMC9463790; doi:10.1186/s12885-022-10060-1)

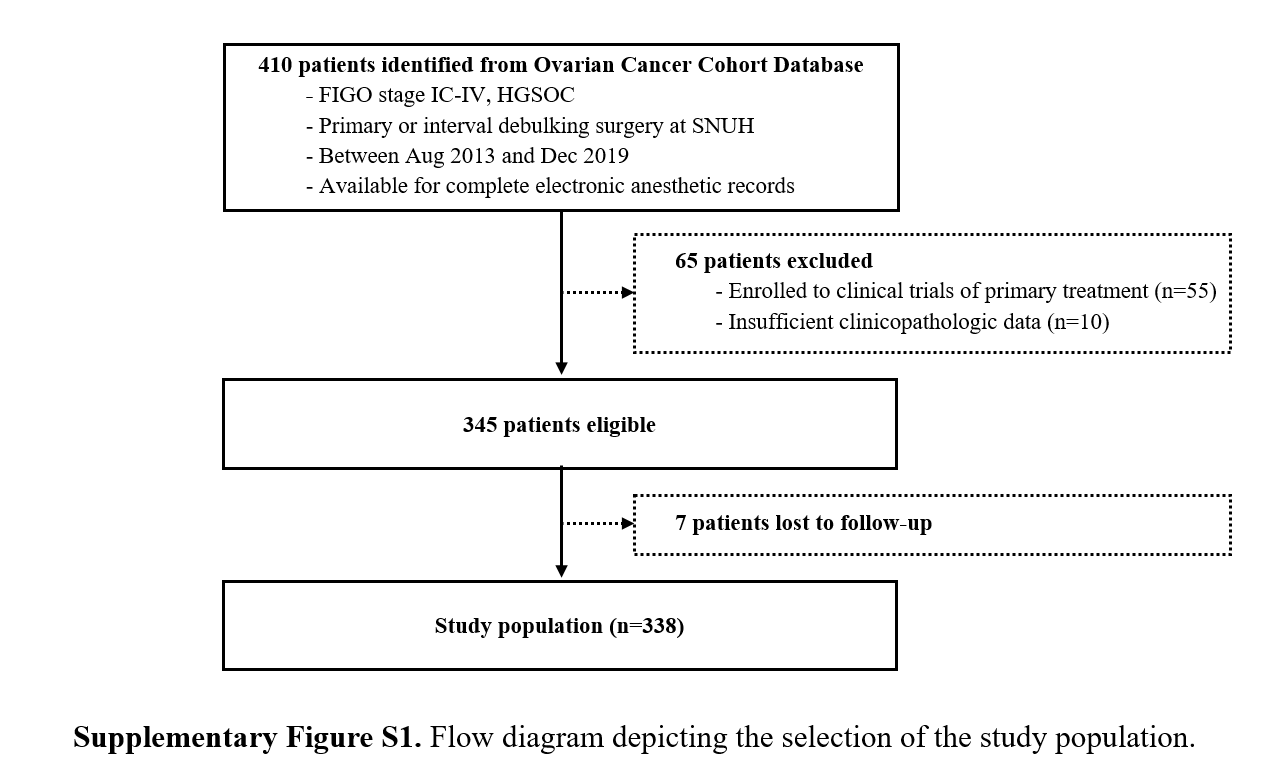

Supplement: Supplementary file 1 — Additional file 1: Supplementary Fig. S1. Flow diagram depicting the selection of the study population. [file 12885_2022_10060_MOESM1_ESM.tif]
