# Supplementary material for: Impact of hemodynamic instability during cytoreductive surgery on survival in high-grade serous ovarian carcinoma
Source: BMC Cancer. 2022 Sep 9;22:965. doi: 10.1186/s12885-022-10060-1 (PMC9463790; doi:10.1186/s12885-022-10060-1)
Supplement: Supplementary file 2 — Additional file 2: Supplementary Table S1. Clinicopathologic characteristics in patients classified according to the cumulative duration of MAP <65 mmHg. [file 12885_2022_10060_MOESM2_ESM.docx]

| **Supplementary Table S1.** Clinicopathologic characteristics in patients classified according to the cumulative duration of MAP <65 mmHg | | | |  |
| --- | --- | --- | --- | --- |
| Characteristics | <30 min of MAP under 65 mmHg  (n=231, %) | ≥30 min of MAP under 65 mmHg  (n=107, %) | *P* |  |
| ***At the time of diagnosis*** |  |  |  |  |
| FIGO stage |  |  | 0.167 |  |
| IC | 16 (6.9) | 3 (2.8) |  |  |
| II | 23 (10.0) | 6 (5.6) |  |  |
| III | 115 (49.8) | 54 (50.5) |  |  |
| IV | 77 (33.3) | 44 (41.1) |  |  |
| Initial serum CA-125 ^a^, IU/ml |  |  |  |  |
| Median (IQR) | 794.5 (215.0−2137.5) | 1438.0 (409.0−2946.0) | 0.012 |  |
| Primary treatment strategy |  |  | 0.955 |  |
| Primary debulking surgery | 154 (66.7) | 71 (66.4) |  |  |
| Neoadjuvant chemotherapy | 77 (33.3) | 36 (33.6) |  |  |
| ***At the time of surgery*** |  |  |  |  |
| Age, years |  |  |  |  |
| Mean ± SD | 56.4 ± 10.4 | 60.9 ± 12.1 | 0.001 |  |
| BMI, kg/m^2^ |  |  |  |  |
| Median (IQR) | 23.1 (20.7−25.1) | 23.5 (21.2−25.8) | 0.096 |  |
| Underweight (<18.5) | 19 (8.2) | 5 (4.7) | 0.347 |  |
| Normal (18.5−22.9) | 96 (41.6) | 38 (35.5) |  |  |
| Overweight (23.0−24.9) | 53 (22.9) | 28 (26.2) |  |  |
| Obesity (≥25.0) | 63 (27.3) | 36 (33.6) |  |  |
| Comorbidities |  |  |  |  |
| Hypertension | 36 (15.6) | 22 (20.6) | 0.259 |  |
| Diabetes | 8 (3.5) | 11 (10.3) | 0.011 |  |
| Liver disease | 7 (3.0) | 0 | 0.102 |  |
| Heart disease | 6 (2.6) | 3 (2.8) | >0.999 |  |
| Renal disease | 0 | 2 (1.9) | >0.999 |  |
| Vascular disease | 1 (0.4) | 1 (0.9) | >0.999 |  |
| Neurologic disease | 2 (0.9) | 3 (2.8) | 0.331 |  |
| Asthma | 2 (0.9) | 0 | >0.999 |  |
| ASA classification |  |  | 0.242 |  |
| 1 | 48 (20.8) | 17 (15.9) |  |  |
| 2 | 151 (65.4) | 69 (64.5) |  |  |
| 3 | 32 (13.9) | 20 (18.7) |  |  |
| 4 | 0 | 1 (0.9) |  |  |
| Surgical complexity score |  |  |  |  |
| Median (IQR) | 5 (4−8) | 8 (5−12) | <0.001 |  |
| Low (≤3) | 30 (13.0) | 3 (2.8) | <0.001 |  |
| Intermediate (4−7) | 129 (55.8) | 46 (43.0) |  |  |
| High (≥8) | 72 (31.2) | 58 (54.2) |  |  |
| Residual tumor after surgery |  |  | 0.771 |  |
| Complete cytoreduction (R0) | 176 (76.2) | 77 (72.0) |  |  |
| <1 cm | 32 (13.9) | 16 (15.0) |  |  |
| 1−2 cm | 13 (5.6) | 9 (8.4) |  |  |
| ≥2 cm | 10 (4.3) | 5 (4.7) |  |  |
| Abbreviations: ASA, American Society of Anesthesiologists; BMI, body mass index; CA-125, cancer antigen 125; FIGO, International Federation of Gynecology and Obstetrics; IQR, interquartile range; MAP, mean arterial blood pressure; SD, standard deviation.  Missing data: ^a^ 3. | | | | |
