# Supplementary material for: Impact of hemodynamic instability during cytoreductive surgery on survival in high-grade serous ovarian carcinoma
Source: BMC Cancer. 2022 Sep 9;22:965. doi: 10.1186/s12885-022-10060-1 (PMC9463790; doi:10.1186/s12885-022-10060-1)
Supplement: Supplementary file 3 — Additional file 3: Supplementary Table S2. Perioperative characteristics in patients classified according to the cumulative duration of MAP <65 mmHg, median performance error, and wobble. [file 12885_2022_10060_MOESM3_ESM.docx]

| **Supplementary Table S2.** Perioperative characteristics in patients classified according to the cumulative duration of MAP <65 mmHg, median performance error, and wobble | | | | | | | | | |
| --- | --- | --- | --- | --- | --- | --- | --- | --- | --- |
| Variables | <30 min of MAP under 65 mmHg  (n=231, %) | ≥30 min of MAP under 65 mmHg  (n=107, %) | *P* | MDPE ≥-4.0%  (n=155, %) | MDPE <-4.0%  (n=183, %) | *P* | Wobble <7.5%  (n=179, %) | Wobble ≥7.5%  (n=159, %) | *P* |
| Preoperative Hb, g/dl | 12.0 ± 1.4 | 11.5 ± 1.4 | 0.003 | 11.8 ± 1.3 | 11.9 ± 1.4 | 0.475 | 12.0 ± 1.4 | 11.6 ± 1.3 | 0.009 |
| Anesthesia time, h | 5.4 (4.2−7.0 | 6.7 (5.1−9.0) | <0.001 | 5.5 (4.3−7.4) | 6.0 (4.6−7.7) | 0.187 | 6.0 (4.9−7.4) | 5.6 (4.3−7.8) | 0.381 |
| Operative time, h | 4.3 (3.1−5.8) | 5.5 (4.0−7.7) | <0.001 | 4.4 (3.2−6.3) | 4.9 (3.5−6.5) | 0.283 | 5.0 (3.6−6.3) | 4.6 (3.2−6.5) | 0.440 |
| Urine output ^a^, ml | 400 (221.3−650) | 510 (300−900) | 0.002 | 400 (230−702.5) | 470 (250−765) | 0.434 | 450 (230−670) | 400 (250−797.5) | 0.701 |
| Estimated blood loss ^b^, ml | 675 (400−1500) | 1500 (800−2700) | <0.001 | 800 (400−1800) | 975 (500−1950) | 0.158 | 900 (500−1800) | 900 (475−1900) | 0.938 |
| RBC transfusion, pack | 1 (0−2) | 3 (1−6) | <0.001 | 1 (0−4) | 2 (0−3) | 0.333 | 1 (0−3) | 1 (0−4) | 0.184 |
| Fluid infusion ^c^, ml | 3100 (1922.5−5400) | 4950 (3425−7475) | <0.001 | 3350 (2000−5612.5) | 3950 (2550−5950) | 0.070 | 3900 (2575−5600) | 3750 (2025−6250) | 0.876 |
| Colloid | 0 (0-500) | 500 (0−1000) | <0.001 | 450 (0−750) | 200 (0−700) | 0.774 | 200 (0−600) | 500 (0−900) | 0.566 |
| Crystalloid ^c^ | 2775 (1800−4662.5) | 4300 (3162.5−6775) | <0.001 | 3225 (1775−4837.5) | 3500 (2350−5300) | 0.038 | 3500 (2225−4800) | 3400 (1750−5400) | 0.744 |
| Presented as mean ± SD or median (IQR).  Abbreviations: Hb, hemoglobin; SD, standard deviation; RBC, red blood cell; IQR, interquartile range; MAP, mean arterial blood pressure; MDPE, median performance error.  Missing data: ^a^ 23; ^b^ 21; ^c^ 12. | | | | | | | | | |
