# Supplementary material for: Impact of hemodynamic instability during cytoreductive surgery on survival in high-grade serous ovarian carcinoma
Source: BMC Cancer. 2022 Sep 9;22:965. doi: 10.1186/s12885-022-10060-1 (PMC9463790; doi:10.1186/s12885-022-10060-1)
Supplement: Supplementary file 4 — Additional file 4: Supplementary Table S3. Factors associated with patients’ overall survival. [file 12885_2022_10060_MOESM4_ESM.docx]

| **Supplementary Table S3.** Factors associated with patients’ overall survival | | | | | | | | | | |
| --- | --- | --- | --- | --- | --- | --- | --- | --- | --- | --- |
| Variables | *Univariate analysis* | | *Multivariate analysis* | | | | | | | |
|  | HR (95% CI) | *P* | aHR (95% CI) | *P* | aHR (95% CI) | *P* | aHR (95% CI) | *P* | aHR (95% CI) | *P* |
| FIGO stage |  |  |  |  |  |  |  |  |  |  |
| III-IV (n=290) vs. I-II (n=48) | 2.681 (0.641−11.207) | 0.177 |  |  |  |  |  |  |  |  |
| Neoadjuvant chemotherapy |  |  |  |  |  |  |  |  |  |  |
| Yes (n=113) vs. No (n=225) | 2.754 (1.399−5.422) | 0.003 | 3.048 (1.531−6.069) | 0.002 | 2.887 (1.459−5.711) | 0.002 | 2.775 (1.391−5.538) | 0.004 | 2.875 (1.449−5.702) | 0.003 |
| Operative time, h |  |  |  |  |  |  |  |  |  |  |
| ≥5.0 (n=161) vs. <5.0 (n=177) | 1.035 (0.526−2.037) | 0.920 |  |  |  |  |  |  |  |  |
| Residual tumor after surgery |  |  |  |  |  |  |  |  |  |  |
| Residual (n=85) vs. R0 (n=253) | 3.372 (1.716−6.629) | <0.001 | 3.609 (1.827−7.129) | <0.001 | 3.578 (1.816−7.050) | <0.001 | 3.642 (1.843−7.196) | <0.001 | 3.529 (1.790−6.959) | <0.001 |
| MAP under 65 mmHg, min |  |  |  |  |  |  |  |  |  |  |
| Continuous (n=338) | 1.003 (0.994−1.011) | 0.543 |  |  |  |  |  |  |  |  |
| ≥30.0 (n=107) vs. <30.0 (n=231) | 1.341 (0.669−2.687) | 0.408 | 1.411 (0.696−2.857) | 0.339 |  |  |  |  |  |  |
| MDPE (%) |  |  |  |  |  |  |  |  |  |  |
| Continuous (n=338) | 0.979 (0.949−1.010) | 0.185 |  |  |  |  |  |  |  |  |
| <-4.0 (n=183) vs. ≥-4.0 (n=155) | 1.510 (0.755−3.019) | 0.244 |  |  | 1.482 (0.740−2.966) | 0.267 |  |  |  |  |
| Wobble (%) |  |  |  |  |  |  |  |  |  |  |
| Continuous (n=338) | 1.137 (0.983−1.315) | 0.083 |  |  |  |  | 1.108 (0.957−1.282) | 0.171 |  |  |
| ≥7.5 (n=159) vs. <7.5 (n=179) | 1.654 (0.835−3.280) | 0.149 |  |  |  |  |  |  | 1.513 (0.762−3.008) | 0.237 |
| Abbreviations: aHR, adjusted hazard ratio; BMI, body mass index; CI, confidence interval; FIGO, International Federation of Gynecology and Obstetrics; HR, hazard ratio; MAP, mean arterial blood pressure; MDPE, median performance error; R0, complete cytoreduction. | | | | | | | | | | |
