# Supplementary material for: Impact of hemodynamic instability during cytoreductive surgery on survival in high-grade serous ovarian carcinoma
Source: BMC Cancer. 2022 Sep 9;22:965. doi: 10.1186/s12885-022-10060-1 (PMC9463790; doi:10.1186/s12885-022-10060-1)
Supplement: Supplementary file 5 — Additional file 5: Supplementary Table S4. Clinicopathologic characteristics in patients classified according to the median performance error. [file 12885_2022_10060_MOESM5_ESM.docx]

| **Supplementary Table S4.** Clinicopathologic characteristics in patients classified according to the median performance error | | | |  |
| --- | --- | --- | --- | --- |
| Characteristics | MDPE ≥-4.0%  (n=155, %) | MDPE <-4.0%  (n=183, %) | *P* |  |
| ***At the time of diagnosis*** |  |  |  |  |
| FIGO stage |  |  | 0.943 |  |
| IC | 9 (5.8) | 10 (5.5) |  |  |
| II | 13 (8.4) | 16 (8.7) |  |  |
| III | 80 (51.6) | 89 (48.6) |  |  |
| IV | 53 (34.2) | 68 (37.2) |  |  |
| Initial serum CA-125 ^a^, IU/ml |  |  |  |  |
| Median (IQR) | 821.5 (243.9−2250.5) | 942.0 (308.5−2535.5) | 0.252 |  |
| Primary treatment strategy |  |  | 0.265 |  |
| Primary debulking surgery | 108 (69.7) | 117 (63.9) |  |  |
| Neoadjuvant chemotherapy | 47 (30.3) | 66 (36.1) |  |  |
| ***At the time of surgery*** |  |  |  |  |
| Age, years |  |  |  |  |
| Mean ± SD | 55.8 ± 11.5 | 59.5 ± 10.6 | 0.002 |  |
| BMI, kg/m^2^ |  |  |  |  |
| Mean ± SD | 22.6 ± 3.1 | 24.0 ± 3.8 | 0.001 |  |
| Underweight (<18.5) | 12 (7.7) | 12 (6.6) | 0.002 |  |
| Normal (18.5−22.9) | 76 (49.0) | 58 (31.7) |  |  |
| Overweight (23.0−24.9) | 36 (23.2) | 45 (24.6) |  |  |
| Obesity (≥25.0) | 31 (20.0) | 68 (37.2) |  |  |
| Comorbidities |  |  |  |  |
| Hypertension | 25 (16.1) | 33 (18.0) | 0.644 |  |
| Diabetes | 8 (5.2) | 11 (6.0) | 0.735 |  |
| Liver disease | 5 (3.2) | 2 (1.1) | 0.254 |  |
| Heart disease | 3 (1.9) | 6 (3.3) | 0.516 |  |
| Renal disease | 1 (0.6) | 1 (0.5) | >0.999 |  |
| Vascular disease | 0 | 2 (1.1) | 0.502 |  |
| Neurologic disease | 3 (1.9) | 2 (1.1) | 0.664 |  |
| Asthma | 0 | 2 (1.1) | 0.502 |  |
| ASA classification |  |  | 0.461 |  |
| 1 | 34 (21.9) | 31 (16.9) |  |  |
| 2 | 100 (64.5) | 120 (65.6) |  |  |
| 3 | 21 (13.5) | 31 (16.9) |  |  |
| 4 | 0 | 1 (0.5) |  |  |
| Surgical complexity score |  |  |  |  |
| Median (IQR) | 6 (4−9) | 6 (4−9) | 0.185 |  |
| Low (≤3) | 20 (12.9) | 13 (7.1) | 0.169 |  |
| Intermediate (4−7) | 80 (51.6) | 95 (51.9) |  |  |
| High (≥8) | 55 (35.5) | 75 (41.0) |  |  |
| Residual tumor after surgery |  |  | 0.610 |  |
| Complete cytoreduction (R0) | 114 (73.5) | 139 (76.0) |  |  |
| <1 cm | 26 (16.8) | 22 (12.0) |  |  |
| 1−2 cm | 9 (5.8) | 13 (7.1) |  |  |
| ≥2 cm | 6 (3.9) | 9 (4.9) |  |  |
| Abbreviations: ASA, American Society of Anesthesiologists; BMI, body mass index; CA-125, cancer antigen 125; FIGO, International Federation of Gynecology and Obstetrics; IQR, interquartile range; MDPE, median performance error; SD, standard deviation.  Missing data: ^a^ 3. | | | | |
