# Supplementary material for: Impact of hemodynamic instability during cytoreductive surgery on survival in high-grade serous ovarian carcinoma
Source: BMC Cancer. 2022 Sep 9;22:965. doi: 10.1186/s12885-022-10060-1 (PMC9463790; doi:10.1186/s12885-022-10060-1)
Supplement: Supplementary file 6 — Additional file 6: Supplementary Table S5. Clinicopathologic characteristics in patients classified according to wobble. [file 12885_2022_10060_MOESM6_ESM.docx]

| **Supplementary Table S5.** Clinicopathologic characteristics in patients classified according to wobble | | | |  |
| --- | --- | --- | --- | --- |
| Characteristics | Wobble <7.5%  (n=179, %) | Wobble ≥7.5%  (n=159, %) | *P* |  |
| ***At the time of diagnosis*** |  |  |  |  |
| FIGO stage |  |  | 0.936 |  |
| IC | 10 (5.6) | 9 (5.7) |  |  |
| II | 17 (9.5) | 12 (7.5) |  |  |
| III | 89 (49.7) | 80 (50.3) |  |  |
| IV | 63 (35.2) | 58 (36.5) |  |  |
| Initial serum CA-125 ^a^, IU/ml |  |  |  |  |
| Median (IQR) | 793.0 (276.2−2399.0) | 973.0 (296.0−2587.3) | 0.293 |  |
| Primary treatment strategy |  |  | 0.012 |  |
| Primary debulking surgery | 130 (72.6) | 95 (59.7) |  |  |
| Neoadjuvant chemotherapy | 49 (27.4) | 64 (40.3) |  |  |
| ***At the time of surgery*** |  |  |  |  |
| Age, years |  |  |  |  |
| Mean ± SD | 54.9 ± 10.9 | 61.1 ± 10.5 | <0.001 |  |
| BMI, kg/m^2^ |  |  |  |  |
| Median (IQR) | 23.1 (20.9−25.3) | 23.4 (20.9−25.6) | 0.977 |  |
| Underweight (<18.5) | 11 (6.1) | 13 (8.2) | 0.838 |  |
| Normal (18.5−22.9) | 74 (41.3) | 60 (37.7) |  |  |
| Overweight (23.0−24.9) | 43 (24.0) | 38 (23.9) |  |  |
| Obesity (≥25.0) | 51 (28.5) | 48 (30.2) |  |  |
| Comorbidities |  |  |  |  |
| Hypertension | 26 (14.5) | 32 (20.1) | 0.173 |  |
| Diabetes | 4 (2.2) | 15 (9.4) | 0.004 |  |
| Liver disease | 6 (3.4) | 1 (0.6) | 0.126 |  |
| Heart disease | 4 (2.2) | 5 (3.1) | 0.740 |  |
| Renal disease | 1 (0.6) | 1 (0.6) | >0.999 |  |
| Vascular disease | 0 | 2 (1.3) | 0.221 |  |
| Neurologic disease | 2 (1.1) | 3 (1.9) | 0.669 |  |
| Asthma | 1 (0.6) | 1 (0.6) | >0.999 |  |
| ASA classification |  |  | 0.757 |  |
| 1 | 33 (18.4) | 32 (20.1) |  |  |
| 2 | 116 (64.8) | 104 (65.4) |  |  |
| 3 | 29 (16.2) | 23 (14.5) |  |  |
| 4 | 1 (0.6) | 0 |  |  |
| Surgical complexity score |  |  |  |  |
| Median (IQR) | 6 (4−9) | 6 (4−10) | 0.786 |  |
| Low (≤3) | 18 (10.1) | 15 (9.4) | 0.722 |  |
| Intermediate (4−7) | 89 (49.7) | 86 (54.1) |  |  |
| High (≥8) | 72 (40.2) | 58 (36.5) |  |  |
| Residual tumor after surgery |  |  | 0.811 |  |
| Complete cytoreduction (R0) | 135 (75.4) | 118 (74.2) |  |  |
| <1 cm | 27 (15.1) | 21 (13.2) |  |  |
| 1−2 cm | 10 (5.6) | 12 (7.5) |  |  |
| ≥2 cm | 7 (3.9) | 8 (5.0) |  |  |
| Abbreviations: ASA, American Society of Anesthesiologists; BMI, body mass index; CA-125, cancer antigen 125; FIGO, International Federation of Gynecology and Obstetrics; IQR, interquartile range; SD, standard deviation.  Missing data: ^a^ 3. | | | | |
